# Mast Cells and Acupuncture Analgesia

**DOI:** 10.3390/cells11050860

**Published:** 2022-03-02

**Authors:** Yingchen Li, Yi Yu, Yuhang Liu, Wei Yao

**Affiliations:** Shanghai Key Laboratory of Acupuncture Mechanism and Acupoint Function, Department of Aeronautics and Astronautics, Fudan University, 220 Handan Road, Shanghai 200433, China; 20110290014@fudan.edu.cn (Y.L.); 17110290008@fudan.edu.cn (Y.Y.); 21210290018@m.fudan.edu.cn (Y.L.)

**Keywords:** mast cell, acupuncture analgesia, acupoint sensitization, mechanical stimuli, TRPV channels, system biology model

## Abstract

Mast cells are widely distributed in various parts of the human body and play a vital role in the progression of many diseases. Recently, the close relationship between mast cells and acupoints was elucidated, and the role of mast cells in acupuncture analgesia has attracted the attention of researchers worldwide. Using mast cells, acupuncture analgesia and acupoint as key words to search CNKI, PubMed, Web of Science and other databases, combining the representative articles in these databases with the published research papers of our group, we summarized: The enrichment of mast cells and the dense arrangement of collagen fibers, microvessels, and nerves form the basis for acupoints as the reaction sites of acupuncture; acupuncture can cause the deformation of collagen fibers and activate TRPV channels on mast cells membrane, so as to stimulate mast cells to release bioactive substances and activate nerve receptors to generate analgesic effect; system biology models are set up to explain the quantitative process of information initiation and transmission at acupuncture points, and indicate that the acupuncture effect depends on the local mast cells density. In a conclusion, this review will give a scientific explanation of acupuncture analgesia from the material basis of acupoints, the local initiation, and afferent biological mechanism.

## 1. Introduction

Mast cells, important immune cells widespread in various areas of the human body, play a vital role in the progression of many diseases. Mast cells were previously thought to cause allergic reaction by releasing cytokines, chemokines, proteases, and biogenic amines after activation. At present, mast cells are also considered to be related to protective host immunity, acting as the sentry of innate immunity and the regulator of adaptive immunity [1,2]. Recently, the close relationship between mast cells and acupoints has been elucidated [3]. The migration, the aggregation, and the activation of mast cells under acupuncture stimuli have been reported [3,4,5].

The efficacy of acupuncture analgesia is recognized worldwide. Acupuncture (punching a tiny needle into the skin and giving mechanical stimulations manually) can relieve the pain caused by many diseases. The effect can be evaluated through observation of animal behaviors, typically the tail flick and the paw withdraw. Zhang et al. observed an increase of pain threshold (PT) after acupuncture at zusanli acupoint (ST36) in adjuvant arthritis (AA) rat models [3]. The acupuncture effect is a complex process, which involves multiple physiological systems from the periphery to the central. Acupoint is the reaction point of disease, and also the stimulation point of acupuncture treatment. Acupoints are enriched in mast cells [4]. Zhu proposed that the local formation of “acupoint sensitization pools” induces a pathological reaction process of “neuropeptide-mast cell-sensitizer release” [6]. Acupoint sensitization is the acupoint transition from its physiological “resting state” to the pathological “active state”. Various sensitization phenomena occur after this transition, including the feelings of acid, swelling, itching, numbness, and pain. These feelings make people involuntarily seek local stimuli such as friction, scratching, pinching, and heat. This process initializes homeostasis regulation and activates the cascade reaction. By promoting homeostasis regulation, acupuncture and moxibustion cure the disease.

The contribution of mast cells in acupuncture analgesia was gradually revealed and became a hotspot, with the proposal of the mechanical signal transduction theory, the humoral theory, and the nerve-humoral theory [7]. The mechanical signal transduction assumes that mast cells can react to mechanical force signals, acting on them through the extracellular matrix. Later, the finding of mechanical sensitive channels on mast cells laid the material basis for theoretical foresight [8,9,10]. Humoral theory means that the active substances released by mast cell degranulation diffuse along the meridian channel through tissue fluid, causing mast cell degranulation. Nerve-humoral theory holds that the active substances released by mast cells can also stimulate nerve endings, enter the center through afferent nerve, and then act on target organs, effectors, or endocrine glands through pituitary or autonomic nerves. Nowadays, it is found that acupuncture can activate mast cells and cause their degranulation (releasing biological substances), inducing an analgesic effect [11,12,13]. With a comprehensive understanding of the previous theories, it is possible to build a bottom-up model and simulate the calcium and mediator signals in the whole system/network, including mast cell degranulation and mast cell-nerve interactions [14,15,16]. To summarize, the effect of mast cells on acupuncture analgesia may bring new insights to the mechanism of acupuncture therapy.

Focusing on the function of mast cells in acupoints, this paper reviews the mechanism of acupuncture analgesia by discussing the following topics: (1) mast cell degranulation and its function, (2) the relationship between mast cells and acupoint sensitization, (3) the physiological responses of mast cells under mechanical stimulations, (4) the synergistic effect of mast cells with collagen fibers and nerves during acupuncture, and (5) the system biology model to quantitatively explain acupuncture information initiation and transmission.

## 2. Characteristics of Mast Cells and Its Function

### 2.1. Origin and Distribution of Mast Cells

Mast cells were discovered by von Recklinghausen in 1863 [17]. Afterwards, Paul Ehrlich detailly described the histological observations of this granules contained cell, and gave it the name [18]. Since then, tremendous progress has been made in the research on the origin, the distribution, and the function of mast cells.

Mast cells are originated from hematopoietic cells in the bone marrow. They migrate into peripheral tissues and differentiate into mature mast cells under the influence of various factors such as monocyte chemoattractant protein-1 (MCP-1), stem cell factor (SCF), transforming growth factor type-β (TGF-β), activin (ACT), etc [19]. Furthermore, the adhesion and the migration of mast cells are influenced by integrin and cytoskeleton, etc. [20,21]. According to the surrounding tissue, mast cells are classified into the mucosal mast cell (granules containing abundant tryptase) and the connective tissue-type mast cell (granules containing tryptase and chymotrypsin) [22].

**Figure 1:**
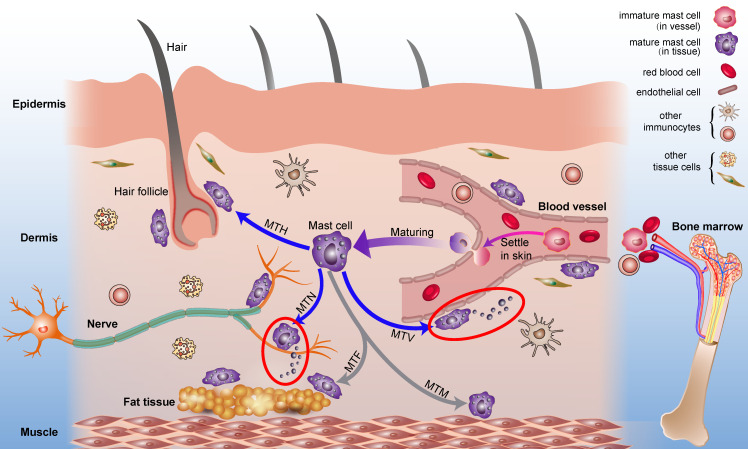
Mast cells in the skin. Mast cell progenitors originate from bone marrow. Under certain conditions, immature mast cells (colored in pink) migrate into peripheral tissues and settle down, mainly in the dermis. Mature mast cells (colored in purple) then migrate to vessels (MTV), nerves (MTN), hair follicles (MTH), muscle tissues (MTM), and adipose tissues (MTA). Mast cells modulate the neighboring cell behaviors by releasing multiple mediators, typically by degranulation after stimulation (marked in red circles), for example, mast cells distributed along vessels can increase vascular permeability, and mast cells distributed along nerves can active nerves. Adapted from [28].

Mast cells are widely distributed in connective tissues and mucosal layers, especially at the interface of the inner and the outer environments such as the skin [23], the digestive tract [24], the airways [25], and other borders where the interaction with the external environment occurs. Besides, mast cells can also be found in organs such as the heart, the liver, and the lung [26]. This paper focuses on mast cells at acupoints, which are distributed in the connective tissue of the skin. Mast cells in the skin are often scattered on the intima, perimysium, and adventitia of the nerve tract; they also distribute around small blood vessels, hair follicles and sweat glands [5,23]. Moreover, mast cells are closely associated with fibroblasts, vascular endothelial cells, cardiomyocytes, etc. A “transgranulation” cellular behavior was also reported [27]. Figure 1 shows the origin and the distribution of mast cells in the skin [28]. Existing in a complex stromal environment, mast cells play a vital role in the circulatory, the neurological, the endocrine, and the immune systems. Like a linking node in a network, mast cells communicate with these physiological systems and couple them together. This review aims to elucidate in detail the mechanism of mast cells’ involvement in acupuncture analgesia.

### 2.2. Mast Cell Degranulation and Its Function

The function of mast cells is mainly fulfilled by the degranulation process. Under degranulation, the cell is activated, and the cell membrane breaks, releasing rich mediators, including histamine, platelet activating factor (PAF), interleukins (IL-1, IL-13, IL-4, and IL-5 etc.), prostaglandin D2 (PGD2), substance P, tryptase, serotonin, bradykinin, heparin, chemokines, and so on [29]. Thanks to the distribution of mast cells, these mediators will quickly act on the neighboring nerves, blood vessels, and muscles, forming a neural-endocrine-immune network. For example, the histamine and platelet activating factor will cause the relaxation of blood vessels and increase the capillary permeability, leukotriene can cause smooth muscle contraction and vasodilation, interleukins work as pro-inflammatory and inflammatory cytokines that activate and recruit inflammatory cells, including granulocytic leukocytes (neutrophils, basophils, eosinophils) and agranular leukocytes (monocytes, lymphocytes) to the site of inflammation, contributing to the development of allergic diseases such as allergic rhinitis. Histamine, substance P, and serotonin etc. can regulate nerve activities.

Mast cells are rich in receptors and in responses to a wide range of stimuli. This allows mast cells to function differently in the presence of different stimuli, having both positive and negative effects in the body depending on specific conditions [30,31]. The activation in the physiological state and body repair state will adjust the homeostasis of the internal environment, which is beneficial to body health by defending against the entering viruses and bacteria. Abnormal activation in a pathological state will cause body discomfort and threaten health. That is, in the case of weak responses, mast cells can aggravate local pathological manifestations [32,33,34]. For example, interleukin-33 (IL-33), produced by epithelial cells, could stimulate mast cells to secrete histamine, which is the main mediator that stimulates nasal rubbing and sneezing in ovalbumin (OVA) induced allergic rhinitis (AR) [35]. Another well-known example of this is the reports that mast cells are activated by COVID-19 and lead to inflammation and fibrosis in the lung. The virus causes mast cells to release pro-inflammatory molecules, thereby contributing to SARS-CoV-2 infection [36,37]; in other settings, especially in severe cutaneous hypersensitivity, mast cells may suppress the process, in part by producing interleukin-10 [38,39]. Mast cells in acupoint sensitization and acupuncture analgesia may reflect their negative and positive effects, respectively.

### 2.3. Mast Cells and Acupoint Sensitization

**Figure 2:**
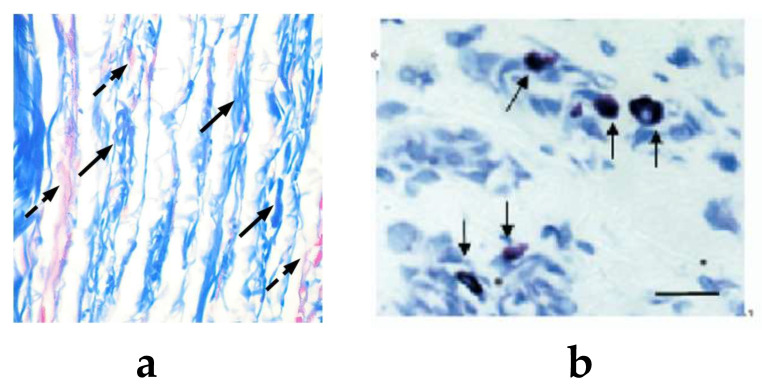
The structure of zusanli acupoint in rats. (**a**) The mallory staining of zusanli acupoint; collagen fibers are in blue, indicated by black solid line arrows. Note that collagen fibers are rich and have a parallel arrangement at the acupoint. Myofibers and blood cells are in red, indicated by black dotted arrows. (**b**) The toluidine blue staining of zusanli acupoint. The scale bar is 10 μm; mast cells are in blue, indicated by solid line arrows. Note that mast cells gather in large numbers at the acupoint [3,43].

The conception of acupoint is introduced in Traditional Chinese Medicine (TCM), as effective targets for acupuncture therapy. Acupoints are a series of special points (about 360 in human) in the skin. These points may become sensitive to mechanical or thermal stimulus under various pathologies [40]. Yuan et al. found that acupoints were mainly collagen fiber-rich areas such as intermuscular connective tissue, peri-neurovascular connective tissue, and organ portal and peri-neural connective tissue [41]. Through magnetic resonance imaging (MRI) and X-ray computed tomography (XCT), Fei et al. also noticed the enrichment of connective tissues in acupoints [42], they found that mast cells, blood vessels, nerves tracts, and lymph vessels, together with the connective tissue as the base, form a very complex structural system. Figure 2 shows the distribution of collagen fibers and mast cells at the zusanli acupoint.

**Figure 3:**
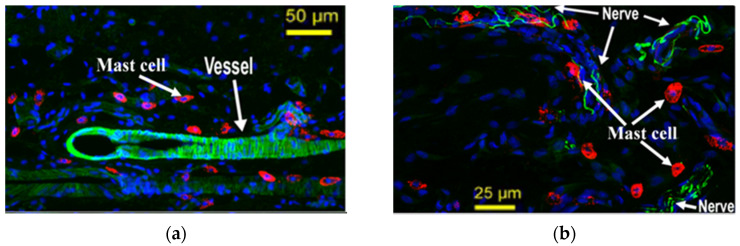
The distribution of mast cells, nerves, and blood vessels at zusanli acupoint in rats after immunofluorescence staining. (**a**) The distribution of mast cells around blood vessels. (**b**) The distribution of mast cells around nerve fibers [28].

In a pioneering histomorphological observation in amputated limbs, Song found that the number of mast cells was significantly larger at acupoints than non-acupoints, she also found that mast cells were located near nerve endings and blood vessels [44,45]. Crivellato’s study gave similar results, finding the abundantly presence of mast cells in the dermal tissue of acupoints area, distributed diffusely or in clusters [18]. Zhang et al. found “synaptic-like” connections between mast cells and nerve endings in the Yang Ming meridian [46,47]. In a histomorphological observation of tissues in acupoints, Luo et al. found a composite strip structure of mast cells, blood vessels, and nerves [4]. The close anatomic relation between mast cells, blood vessels, and nerves implies their reciprocity correlation. Figure 3 shows the distribution of mast cells along blood vessels and nerves of zusanli acupoint in rats.

Acupoints are reaction sites of disease and targets for acupuncture. Many chemicals are involved in acupoint sensitization, they form the so-called “acupoint sensitization pool”. He et al. revealed that the high expression of local allergic substances and nociceptive neuropeptides, such as substance P, calcitonin gene related peptide (CGRP), histamine, serotonin, and tryptase, are responsible for the acupoint sensitization [48]. By releasing these important chemicals, mast cells are closely associated with both the acupoint sensitization and the acupuncture effect [49]. Ding et al. reported that the release of serotonin, histamine, and tryptase during mast cell degranulation regulated the acupoint sensitization [12]. The released substance P may also be involved [50].

Moreover, He et al. found that the concentration of substance P, a calcitonin gene related peptide, at the same acupoint, is different under normal, pathological, and acupuncture conditions [51]. Using high-performance liquid chromatography (HPLC) to measure the adenosine concentration at acupoints, Wang et al. found significant differences before and after modeling and acupuncture [52]. These findings imply a dynamic variation of chemicals at the acupoints, manipulated by the inner environment or the external stimulation. Therefore, a further investigation of the relationship between mast cells and the acupoint sensitization is the key to the mechanism of acupuncture analgesia.

## 3. Activation and Mechanical Sensitivity of Mast Cells

### 3.1. Degranulation of Mast Cells under Mechanical Stimulations

Mast cells are activated by a variety of pathways, such as IgE antibody-antigen complexes, pathogens in the environment, physical stimuli (pressure, heat, electricity, and light), etc. The mechanical sensitivity is one of the main factors for mast cells activation. For example, a mechanical removal of the airway epithelium disrupts the mast cell structure and causes the degranulation, which influences the airway function [25]. Shimbori et al. found that the cyclic mechanical stress induced mast cell degranulation in the rat lung, which contributed to the pulmonary fibrosis [53]. The mechanical sensitivity of mast cells under acupuncture has been widely recognized. Zhang et al. found that the degranulation rate of mast cells increased significantly after the mechanical stimulation of acupuncture [3]. They argued that the released biological mediators would effectively act on the neighboring nerves, blood vessels, and muscles, potentially impacting on the endocrine, the immune, and the neurological systems. In this way, the mechanical stimulation was interpreted into the biological information [54]. Yang et al. found that shear stress induced the calcium changes in rat basophilic leukemia cells (RBL-2H3, a model cell line for mast cells) and led to histamine release [55]. Wang et al. further confirmed the existence of membrane currents during mast cell degranulation under the mechanical stimulation [56]. To conclude, a reasonable assumption is that the acupuncture analgesia effect may begin with the mast cell activation under the mechanical stimulation.

### 3.2. Mechanosensitive Channels of Mast Cells

Mast cell membranes are enriched with receptors and ion channels, including immunoglobulin E receptor (IgE–FcεRI), Toll-like receptors, immunoglobulin receptor (Ig–FcγRIII), stem cell factor receptors, G protein-coupled receptors, the ATP-sensitive receptors, etc [57]. Nowadays, transient receptor potential vanilloid channels have been reported to be responsible for the mechanical sensitivity of mast cells [8,54,55,58]. Besides, the stretch-activated (SA) chlorine channels also play a role [56]. Transient receptor potential vanilloid channels are reported to exist in HMC-1 (human leukemia cells), RBL-2H3 (rat basophils), and other model cells for the in-vitro study of mast cells. Though not identical, these model cells demonstrate the main characteristics of mast cells.

Members of the transient receptor potential vanilloid family consist of TRPV1 to TRPV6, of which TRPV1 to TRPV4 are sensitive to mechanical or thermal stimulation. Transient receptor potential vanilloid channels are activated to induces a calcium flow into the cell [58]. Zhang et al. convinced the expression of TRPV1, TRPV2, and TRPV4 in HMC-1 cells. They also found that the TRPV2 channel can be activated under mechanical, heat, and laser stimulations. Meanwhile, an increase of histamine release was detected. Moreover, the channel currents (measured by a patch clamp) could be inhibited by the transient receptor potential vanilloid specific inhibitor ruthenium red (RuR) [54]. Stokes et al. convinced the existence of TRPV1, 2 and 6 channels in RBL-2H3 cells. They also detected the current flow through the TRPV2 channel under mechanical and thermal stimulations [8]. Yang et al. also observed the increase of the intracellular calcium concentration and the release of histamine when shear stress was applied to RBL-2H3 cells [55]. They reported a participation of the TRPV4 channel. To conclude, the inflow of calcium seems the key to the mechanical activation of mast cells. The transient receptor potential vanilloid channels, expressed in mast cell membranes, are the main receptors and sensors for mechanical stimulation, such as acupuncture.

The intracellular signaling pathways from transient receptor potential vanilloid channels opening to mast cell degranulation remain unclear. The TRPV2- Protein kinase A (PKA)-Calcium-Inositol triphosphate (IP3) pathway may be involved [8,16]. Moreover, the cytoskeleton also plays a role in the mechanical sensitivity of mast cells. Fowlkes et al. found that a mechanical stretching of 3-dimensional cultured RBL-2H3 cells could induce degranulation, but after blocking the RGD-Integrin by Echistatin, the degranulation was significantly inhibited [59]. Stretch-activated chlorine channels are also associated with mast cell degranulation. Wang et al. found that osmotic stress activated stretch-activated chlorine channels in HMC-1 cells, generated membrane currents, and caused cell degranulation. The degranulation could be inhibited by DIDS (a chloride channel blocker) [56]. They presumed that the activation of stretch-activated chlorine channels induced the chlorine influx and caused cell hyperpolarization, then the increased cross-membrane potential drove the calcium inflow and consequently induced the cell degranulation.

## 4. Mast Cells and Acupuncture Analgesia

### 4.1. Mast Cells in Acupuncture Analgesia

Zhang et al. found the increase of pain threshold after acupuncture at zusanli acupoint in adjuvant arthritis rat models depended on mast cell degranulation in the neighboring tissue. After reducing the mast cell degranulation with disodium cromolyn (DSCG, mast cell membrane stabilizer), the analgesic effect of acupuncture was inhabited [3]. Cui et al. found that the analgesic effect was closely related with the intensity of mechanical stimulation, and mast cell deficiency in rat attenuated the analgesic effect of acupuncture [60]. Therefore, they concluded that mast cells were essential in acupuncture analgesia. As discussed previously, the TRPV2 channel on mast cells also affects the analgesic effect of acupuncture. Huang et al. observed a great reduction in mast cell degranulation rate at zusanli acupoint in the TRPV2 gene knockout mice (comparing with wild-type animals), as well as a suppression of acupuncture analgesic effect [61].

Mast cells release multiple kinds of biological substances, some of which may be involved in acupuncture analgesia [5]. Huang et al. found that both histamine injection and acupuncture at zusanli acupoint in adjuvant arthritis rats increased the pain threshold, and also promoted the mast cell degranulation. The pretreatment with clemastine (histamine H1 receptor antagonist) could suppress the analgesic effect of acupuncture and decrease the mast cell degranulation rate induced by histamine, while the degranulation rate induced by acupuncture was not affected. Moreover, the pretreatment with disodium cromolyn reduced the mast cell degranulation in both conditions, but the analgesic effect remained in the histamine injection group. These experiments indicated a key role of histamine in the activation of mast cells and the fulfillment of acupuncture analgesia, with a positive feedback effect [62]. Through microdialysis sampling and high-performance liquid chromatography detection of acupoint tissues, Goldman et al. reported an increase in ATP, ADP, AMP, and adenosine induced by acupuncture. Adenosine could induce the anti-nociceptive effect by activating the adenosine A1 receptor. The anti-nociceptive effect could also be reproduced with a direct injection of an agonist to the receptor. Acupuncture treatment fails to suppress pain in the mice lacking adenosine A1 receptors. These observations indicate that adenosine mediates acupuncture analgesia effects [63].

### 4.2. Function of Mast Cells and Collagen at Acupoint

The clinical criterion for achieving acupuncture effect is the acquisition of sensations (a concept called De Qi in Chinese), which is a feeling, including tingling, numbness, and heaviness, elicited by acupuncture. Acupuncturists feel the needle sink tightly, like a fish swallowing a hook [64]. Researchers are attempting to reveal the biophysical basis of this subjective, vague, and incomprehensible concept. Liu et al. performed a biopsy on a patient with the meridian pathology, and they assumed that De Qi is related with the tubular structure formed by the interconnected collagen fibers [65]. The bundles of collagen fibers arrange in parallel at the zusanli acupoint, having a high transmittance of 9–20 um infrared rays [42]. Based on magnetic resonance imaging and X-ray computed tomography observations of the acupoint, Langevin et al. concluded that De Qi is a manifestation of the mechanical coupling between the subcutaneous collagen fibers and the needle body [66]. Collagen fibers are intertwined and interlaced, forming a three-dimensional network in the connective tissue. In the De Qi state, the mechanical stimulation of the needle (lifting, thrusting, and rotation) effectively causes tissue deformation, and the signal is easily transferred to the mast cell, inducing its degranulation. This hypothesis is supported by animal experiments. Yu et al. destroyed the collagen at zusanli acupoint in rats by collagenase, and they found that acupuncture could not cause mast cell degranulation effectively, thus the analgesic effect was significantly weakened. Moreover, the lifting and twisting force of the needle body on the acupoint was dramatically reduced [43]. Therefore, effective coupling of the needle body to the collagen at the acupoint is the key to De Qi during acupuncture.

### 4.3. Mast Cell-Nerve Cell Interaction at Acupoint

Nerves play an essential role in the acupuncture process. The acupuncture analgesic effect is significantly attenuated by either blocking the peripheral nerves at acupoints, or blocking the nerve pathways, or damaging part of the central nervous system. Zhu et al. suggested that the nerve excitation at acupoints was necessary for acupuncture effect [67]. Sa et al. observed discharges of the peripheral nerve tracts when stimulating the zusanli acupoint in rats. The injection of disodium cromolyn (blocking mast cell degranulation) at the acupoint weakened the discharges [68]. This experiment verified the mast cell participation in the changing of neural electrical signals during acupuncture. The changing of neural electrical signals could also be detected at the dorsal root of the spinal cord [69], indicating the existence of an afferent signal pathway. Yin et al. further proved that the histamine released by mast cell degranulation participated in the activation process of acupuncture neuroelectric signals [70].

Mast cell-nerve cell spatial contacts has been verified both in vitro and in vivo [71]. The functional associations between mast cells and nerves have been proven at both the anatomic and the molecular levels [72,73,74]. The interaction between nerve cells and mast cells is mutual. Immune activation of mast cells by the injection of antigen into sensitized animals causes the release of histamine to excite neurons, which can be inhibited by histamine H2 receptor blockers [75]. The stimulated nerve cells will also affect the activity of mast cells. Studies have found that stimulating the enteric nerve in rats caused histamine release, and reduced mast cell degranulation [76]. Prolonged electrical stimulation of sensory nerves can lead to degranulation of mast cells and an increase of the vascular permeability in the rat [77].

To conclude, mast cells and nerves interact with each other. The mediators released by mast cells induce neuroelectric activities (both locally and centrally), and the transmitters released from sympathetic neurons manipulate mast cell activation in turn, forming a feedback network. One possible advantage of the network, although remaining mysterious, is the capability to link different parts of the body together and cause collaborative responses.

## 5. Mathematical Model of Mast Cell Involvement in Acupuncture Analgesia

The acupoint response to mechanical stimulation includes local mast cell degranulation and the cascade reaction of biological transmitters. The acupuncture is a complex and multi-scale process, involving biochemical and biophysical factors. Mathematical modeling provides an effective method to help systematically understand and quantitatively analyze the process. In this review, we will give a very brief introduction to those models. One of the important advantages of these models is the convenience to giving deductive but reasonable quantitative results that are not possible to measure with current techniques. Yannick et al. analyzed the effect of mast cells density on acupuncture by numerical simulation [78]. Shi et al. established a mathematical model to simulate the intracellular calcium signal and degranulation of a mast cell [79]. Yao et al. proposed a series of mathematical models that demonstrated the biophysical and biochemical processes during acupuncture. The calcium rise in a mast cell was described using differential equations based on behaviors of the ion channels [16], and the calcium signal propagation in mast cells network was investigated [14]. Numerical simulation results showed that the acupuncture effect is not only dependent on the mast cells at the acupoints, but is also influenced by the local mast cell density. The chain reactions of mast cell degranulation and neuroreceptor activation are not elicited where mast cell density is low. The vast majority of acupoints in the human body are enriched in mast cells, so acupuncture at these acupoints is easier in order to produce acupuncture effects. Furthermore, the mast cell and nerve interaction was also modeled mathematically [14,15,16].

**Figure 4:**
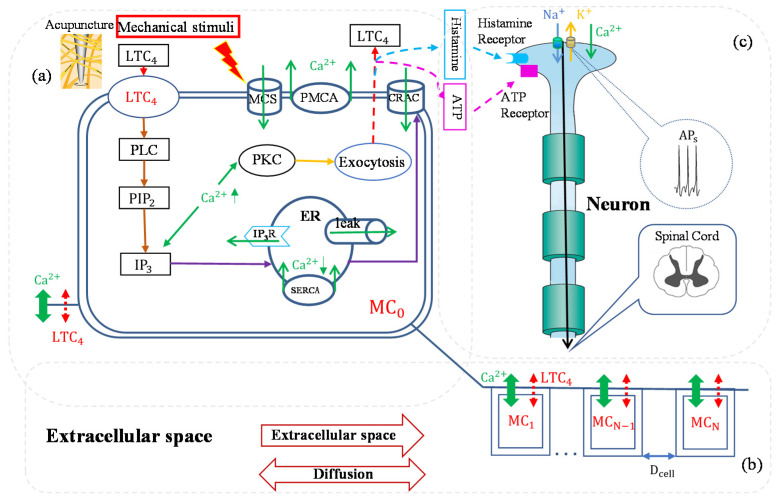
The bio-mathematical model of acupuncture effect by the synergistic action mechanism of interstitial substances. The kinetic models describe the biochemical response of individual cell to stimulation. The conductance models describe the transmission of electrical signals in continuous nerve fibers. The mast cells network models describe the information response between mast cells. (**a**) The representation of single mast cell. (**b**) The representation of mast cells network. (**c**) The representation of nerve cells. Unidirectional arrows represent substance transport directions, the green solid line bidirectional arrows represent calcium, and the red dash line bidirectional arrows represent mediators such as leukotriene c-4 (LTC_4_) and histamine, etc. MC_0_ is the mast cell activated by mechanical stimuli; the steps leading from mechanical sensitivity channel (MSC) activation to calcium release from the calcium store (Endoplasmic Reticulum) into the cytosol and mediators release into extracellular space by exocytosis described in MC_0_. A lane of model mast cells (MC_1_ means the first mast cell from MC_0_ in the flow direction; MC_−__1_ means the first mast cell from MC_0_ in the contra-flow direction; MC*_N_* means the N*th* mast cell from MC_0_ in the flow direction; and MC_−_*_N_* means the N*th* mast cell from MC_0_ in the contra-flow direction) are separated by *D_cell_*. Each cell exchanges biological messengers through cell membrane with extracellular space. Extracellular space is regarded as continuous, and diffusion and convection are included. Adapted from [14].

The second advantage of mathematical models is the ability to synthesize the complex, multiscale process with a framework of combined abstract blocks (or stages). The dynamic process of the mast cell activation is illustrated in Figure 4 [14,15]. In the first stage, mechanical stimulations activate the mechanical sensitive ion channels on mast cells membrane and allow calcium entry; the intracellular calcium increase activates protein kinase C (PKC) and increases the sensitivity of secretory granules to calcium, thus driving exocytosis and mediators release. In the second stage, the released mediators trigger cellular responses through the G-protein linked receptors. These receptors bind to phospholipase C (PLC), and phospholipase C catalyzes the hydrolysis of phosphatidylinosital biphosphate (PIP2) and the release of inositol triphosphate. Inositol triphosphate acts on receptors (IP3R) of the endoplasmic reticulum (ER) and leads the stored calcium release; the depletion of calcium in endoplasmic reticulum triggers calcium entry through calcium release-activated calcium (CRAC) channels. In the third stage, mediators diffuse or flow in extracellular space (ECS) and activate other mast cells. Mediators can also bind to receptors of adjacent nerve terminals (sensory neuron) and trigger action potentials, which induce passive electrical flow from primary sensory neurons to spinal cord neurons.

Table 1 shows the response results of mast cells and nerves. Nerve cells at D_ist_ (distance from mast cell) of 200 μm, 400 μm, 600 μm, and 800 μm can be activated by mediators released from the stimulated mast cell. The response time of membrane potential (E_m_) activation of the nerve cell increases with distance, causing this signal intensity to decrease. However, when the D_ist_ is 1 × 10^−4^ m, the nerve cells are no longer activated, in other words, when the distance between the mast cell and the nerve cell is too large, there is no acupuncture analgesia effect.

## 6. Conclusions and Discussion

Acupuncture analgesia is an internationally accepted effective treatment in Traditional Chinese Medicine and has a wide range of applications. However, the lack of scientific elucidation of the background mechanism has hindered its modern development, as well as its application in mainstream medicine. In this paper, we reviewed the literature on mast cells and acupuncture analgesia, which is a major concern in revealing the acupuncture mechanism. These research efforts in the past decades have contributed to a scientific explanation of acupuncture effect in all aspects: from the material basis of acupoints and acupuncture (in anatomical, cellular, and molecular levels), to the initiation, transformation, and propagation of acupuncture signals. Mast cells play a key role—without doubt.

Acupuncture in a broader context includes mechanical (acupuncture), electrical (electroacupuncture), and heat (moxibustion) treatments. This review only focuses on acupuncture. Because the main mechanical sensitive channel TRPV2 can also be activated by heat stimulation [57], we suppose that the therapeutic mechanism of moxibustion is similar to that of acupuncture: activate mast cells by heat or mechanism stimulation, which leads to an analgesia effect. Some works on moxibustion support this hypothesis [80]. The mechanism of electroacupuncture is complex; electrical stimulation may activate both mast cells and nerve cells. Acupuncture mainly activates the local mast cell, while electroacupuncture not only caused degranulation of mast cells at the zusanli acupoint, but also in the abdominal cavity on the same meridian [81]. On one hand, the effect of electroacupuncture on mast cells and mast cell-mediated analgesia is not as good as that of acupuncture. On the other hand, unlike what was observed in acupuncture, the analgesia effect of electroacupuncture cannot be totally blocked by the mast cell membrane stabilizer (disodium cromolyn) [82]. It reminds us that there is another mechanism involved in electroacupuncture analgesia besides mast cell activation.

## Figures and Tables

**Table 1:** Response time of E_m_ and [Ca^2+^]_i_ (calcium concentration) peak of different D_ist_s [15].

D_ist_	Response Time of E_m_	[Ca^2+^]_i_ Peak (µM)
2 × 10^−5^ m	22 s	0.33
4 × 10^−5^ m	24 s	0.31
6 × 10^−5^ m	27 s	0.29
8 × 10^−5^ m	33.1 s	0.27
1 × 10^−4^ m	No	0.24

## Data Availability

Not applicable.
